# Between evidence and allegation: factual accuracy in discussions on fluoride toxicity and research integrity

**DOI:** 10.1007/s00204-026-04531-8

**Published:** 2026-09-01

**Authors:** Andrea Buettner, Patrick Diel, Gerhard Eisenbrand, Bernd Epe, Petra Först, Tilman Grune, Sabine Guth, Dirk Haller, Volker Heinz, Michael Hellwig, Hans-Ulrich Humpf, Henry Jäger, Sabine E. Kulling, Alfonso Lampen, Marcel Leist, Angela Mally, Doris Marko, Ute Nöthlings, Elke Röhrdanz, Angelika Roth, Joachim Spranger, Stefan Vieths, Wim Wätjen, Jan G. Hengstler

**Affiliations:** 1 Chair of Aroma and Smell Research, Friedrich-Alexander-Universität Erlangen-Nürnberg, Henkestrasse 9, 91054 Erlangen, Germany; 2 Fraunhofer Institute for Process Engineering and Packaging IVV, Giggenhauser Strasse 35, 85354 Freising, Germany; 3 Department of Molecular and Cellular Sports Medicine, Institute of Cardiovascular Research and Sports Medicine, German Sport University Cologne, Am Sportpark Müngersdorf 6, 50933 Cologne, Germany; 4 Kühler Grund, 48/1, 69126 Heidelberg, Germany; 5 Institute of Pharmaceutical and Biomedical Sciences, University of Mainz, 55128 Staudingerweg, Mainz, Germany; 6 Food Process Engineering, TUM School of Life Sciences, Technical University of Munich, Weihenstephaner Berg 1, 85354 Freising, Germany; 7 German Institute of Human Nutrition Potsdam-Rehbrücke (DIfE), Arthur-Scheunert-Allee 114-116, 14558 Nuthetal, Germany; 8 Department of Toxicology, Leibniz Research Centre for Working Environment and Human Factors (IfADo), Ardeystrasse 67, 44139 Dortmund, Germany; 9 Chair of Nutrition and Immunology, Technical University of Munich, Gregor-Mendel-Strasse 2, 85354 Freising, Germany; 10 ZIEL Institute for Food & Health, Technical University of Munich, Weihenstephaner Berg 1, 85354 Freising, Germany; 11 DIL German Institute of Food Technology, Professor-von-Klitzing-Strasse 7, 49610 Quakenbrück, Germany; 12 Chair of Special Food Chemistry, Technical University Dresden, Bergstrasse 66, 01062 Dresden, Germany; 13 Institute of Food Chemistry, Universität Münster, Corrensstrasse 45, 48149 Münster, Germany; 14 University of Natural Resources and Life Sciences, Gregor-Mendel-Strasse 33, Vienna, 1180 Austria; 15 Institute of Applied Biosciences, Karlsruhe Institute of Technology (KIT), Adenauerring 20A, 76131 Karlsruhe, Germany; 16 Risk Assessment Strategies, German Federal Institute for Risk Assessment (BfR), Max‑Dohrn‑Strasse 8‑10, 10589 Berlin, Germany; 17 Division for In Vitro Toxicology and Biomedicine, Department of Biology, University of Konstanz, Universitaetsstrasse 10, 78464 Konstanz, Germany; 18 Department of Toxicology, University of Würzburg, Versbacher Strasse 9, 97078 Würzburg, Germany; 19 Department of Food Chemistry and Toxicology, Faculty of Chemistry, University of Vienna, Währinger Strasse 38, Vienna, 1090 Austria; 20 Institute for Nutrition and Food Science, Rheinische Friedrich-Wilhelms-University Bonn, Fiedrich-Hirzebruch-Allee 7, 53115 Bonn, Germany; 21 Unit Reproductive and Genetic Toxicology, Federal Institute for Drugs and Medical Devices (BfArM), Kurt-Georg-Kiesinger Allee 3, 53175 Bonn, Germany; 22 Charité – Universitätsmedizin Berlin, Charitéplatz 1, 10117 Berlin, Germany; 23 Paul-Ehrlich-Institute, Paul-Ehrlich-Strasse 51-59, 63225 Langen, Germany; 24 Institute of Agricultural and Nutritional Sciences, Martin-Luther-University Halle-Wittenberg, Weinbergweg 22, 06120 Halle (Saale), Germany

In 2020, we published a review in this journal evaluating the potential adverse effects of fluoride (Guth et al. 2020, 2021). After systematically analyzing epidemiological studies, animal experiments, and in vitro data, we concluded that the available evidence does not support the assumption that fluoride exposure at levels typically encountered in Europe causes adverse health effects, including developmental neurotoxicity. Fluoride requires careful risk assessment because it occupies a unique position in public health: controlled exposure is recommended for the prevention of dental caries, whereas excessive exposure should be avoided because of the risk of dental or skeletal fluorosis. At the time, particular attention focused on the question of whether fluoride exposure at common environmental levels might impair neurodevelopment in children. Based on the available evidence, we found no convincing indication that fluoride acts as a developmental neurotoxicant at exposure levels relevant for European populations.

Recently, a publication by Neurath (2025) addressed our work not by engaging with its scientific arguments, but by alleging that the authors, as well as scientific organizations associated with them, were influenced by industry interests. Rather than engaging with the scientific arguments, the article makes a series of demonstrably false factual allegations about the authors and associated scientific organizations, thereby calling their scientific integrity into question without evidence. For example, the senior author of our review (J.G.H.) is accused of having received more than USD 500,000 from the tobacco industry. This allegation is demonstrably false. He has never received funding from the tobacco industry; all research funding received by his group originates from public sources and is publicly disclosed. The article further suggests that the German Research Foundation (DFG) and its Senate Commission on Food Safety (SKLM) serve as instruments through which industry influences scientific assessments. No evidence is provided to support these claims. Because these demonstrably false factual assertions directly impugn the integrity of individual researchers, and undermine confidence in evidence-based risk assessment, we consider it important to correct the record. A detailed point-by-point response is provided in the Supplementary Material.

The broader issue extends beyond the individuals concerned. Different interpretations of the same body of evidence are common in science and risk assessment and should be evaluated on the basis of data and methodology. The situation is fundamentally different when allegations are presented as factual statements. Factual claims are, by definition, verifiable and should therefore be held to the same standards of accuracy as scientific data. Publications in peer-reviewed journals are widely regarded as having undergone editorial and scientific scrutiny. Consequently, factual statements published in the scientific literature may be assumed by readers to have been checked for accuracy. When demonstrably incorrect allegations enter the scientific record, they can be propagated through citations, databases, and secondary reporting, potentially affecting the reputation of researchers and scientific institutions long after the original claims have been shown to be unfounded. Scientific criticism and disagreement are essential components of research. Demonstrably false factual assertions, however, should be corrected once identified.

After publication of our review, subsequent evaluations have reached broadly similar conclusions. Most notably, the European Food Safety Authority (EFSA 2025) conducted a comprehensive re-evaluation of fluoride in 2025, including the large body of studies published after our review. EFSA concluded that the available evidence was insufficient to establish an association between fluoride exposure and adverse neurodevelopmental effects at fluoride concentrations typically encountered in European drinking water supplies. Likewise, the German Federal Institute for Risk Assessment (BfR 2026) stated that, based on the totality of the available evidence, there is no reason to classify fluoride as a neurotoxicant. Both authorities nevertheless emphasized the need for further research to improve our understanding of fluoride kinetics and biological activity.

The question of fluoride toxicity remains a matter for scientific investigation. The question of factual accuracy should not.

## Supplementary Information

Below is the link to the electronic supplementary material.

Supplementary Material 1

## Data Availability

No datasets were generated or analysed during the current study.

## References

[CR1] EFSA Scientific Committee (2025) Updated consumer risk assessment of fluoride in food and drinking water including the contribution from other sources of oral exposure. EFSA J 23(7):e9478. 10.2903/j.efsa.2025.9478 40698337 PMC12280829

[CR2] German Federal Institute for Risk Assessment (BfR) (2026) Fluoride: important for teeth. Frequently Asked Questions (FAQ). BfR, Berlin. Available at: BfR Fluoride: important for teeth. Accessed 6 Jun 2026

[CR3] Guth S, Hüser S, Roth A, Degen G, Diel P, Edlund K, Eisenbrand G, Engel KH, Epe B, Grune T, Heinz V, Henle T, Humpf HU, Jäger H, Joost HG, Kulling SE, Lampen A, Mally A, Marchan R, Marko D, Mühle E, Nitsche MA, Röhrdanz E, Stadler R, van Thriel C, Vieths S, Vogel RF, Wascher E, Watzl C, Nöthlings U, Hengstler JG (2020) Toxicity of fluoride: critical evaluation of evidence for human developmental neurotoxicity in epidemiological studies, animal experiments and in vitro analyses. Arch Toxicol 94(5):1375–1415. 10.1007/s00204-020-02725-2 32382957 PMC7261729

[CR4] Guth S, Hüser S, Roth A, Degen G, Diel P, Edlund K, Eisenbrand G, Engel KH, Epe B, Grune T, Heinz V, Henle T, Humpf HU, Jäger H, Joost HG, Kulling SE, Lampen A, Mally A, Marchan R, Marko D, Mühle E, Nitsche MA, Röhrdanz E, Stadler R, van Thriel C, Vieths S, Vogel RF, Wascher E, Watzl C, Nöthlings U, Hengstler JG (2021) Contribution to the ongoing discussion on fluoride toxicity. Arch Toxicol 95(7):2571–2587. 10.1007/s00204-021-03072-6 34095968 PMC8241794

[CR5] Neurath C (2025) The sugar industry’s efforts to manipulate research on fluoride effectiveness and toxicity: a ninety-year history. Environ Health 24:54. 10.1186/s12940-025-01137-7 41016930 PMC12477810

