# Supplementary material for: Between evidence and allegation: factual accuracy in discussions on fluoride toxicity and research integrity
Source: Arch Toxicol. 2026 Sep 1;100(10):4801–3. doi: 10.1007/s00204-026-04531-8 (PMC13593672; doi:10.1007/s00204-026-04531-8)
Supplement: Supplementary file 1 [file 204_2026_4531_MOESM1_ESM.docx]

*Supplemental Material (electronic supplement)*

**Background:** The text reproduced below was already sent to the editors of *Environmental Health* to correct false statements published in Neurath et al., 2025. As no response was received from the journal’s editors, the present reply was submitted to the Archives of Toxicology.

**Correction of factual inaccuracies and clarification of statements concerning DFG, SKLM, and fluoride toxicity**

**Reply to:**

Neurath C. *The sugar industry's efforts to manipulate research on fluoride effectiveness and toxicity: a ninety-year history.* Environ Health. 2025 Sep 29;24(1):62. doi: 10.1186/s12940-025-01154-x. PMID: 41016930; PMCID: PMC12477810.

**Response to False Factual Allegations**

We regret that recent statements published in *Environmental Health* have contained false statements about the German Research Foundation (DFG) and the Senate Commission on Food Safety (SKLM), as well as incorrect personal allegations against individual scientists. We briefly address these points below and extend an invitation to return the discussion to a scientific level.

**False statements regarding the German Research Foundation (DFG) as well as the Senate Commission on Food Safety (SKLM)**

The article in *Environmental Health* argues that the sugar industry manipulates research on fluoride toxicity and that the DFG and SKLM serve as instruments in this process:

*“…it is amazing how many people who are on the committees at the BfR, DFG or EFSA are in contact with ILSI at the same time. ILSI was founded in 1978 by Coca-Cola, Pepsi Cola, General Foods, Kraft (owned by the Philip Morris tobacco company), and Procter & Gamble.” (Neurath, 2025; p. 22,)*

*“The authors [of a publication of the SKLM on fluoride toxicity] all claim to be independent and declare they have no conflicts of interest. But a look into their history reveals that they are all members or closely associated with a German commission [SKLM] that reviews chemical and food risks but seems to have been co-opted by people connected to an industry front group, the International Life Sciences Institute (ILSI) [263]. ILSI was founded by a vice-president of Coca-Cola and has been funded by Coca-Cola along with a long list of major companies in the sugary foods, processed foods, infant formula, chemical, pesticide, oil, and pharmaceutical industries [264,265].“ (Neurath, 2025; p.18)*

The claim that the DFG, the SKLM, or regulatory agencies such as the German Federal Institute for Risk Assessment (BfR) or EFSA are influenced by, or closely connected to, ILSI, Coca-Cola, Philip Morris, or comparable companies is factually incorrect. Neither the DFG nor the SKLM operates under the sponsorship or direction of commercial entities. The DFG is a self-governing, publicly funded research organisation; the SKLM is an independent scientific commission within the DFG, tasked with assessing current evidence on food-related health risks.

Membership in DFG commissions does not constitute evidence of external influence, nor does it imply alignment with the interests of specific companies. Claims of co-optation require verifiable evidence, which is not provided in Neurath (2025). The scientific independence of both the DFG and the SKLM is further ensured by established conflict of interest regulations and mandatory transparency and disclosure procedures^[1]^ for all commission members and permanent guests. Specifically, members and permanent guests of the Commissions confirm in writing, prior to their first involvement or after extending their membership, their obligation to disclose in a timely manner any circumstances that could constitute a conflict of interest. In addition, participants are asked before each meeting of the Commission whether any conflicts of interest exist. These procedures ensure that any potential conflicts of interest are systematically addressed and properly documented.

*“Are the Guth paper [a publication of the SKLM] authors truly independent? Almost all the 31 co-authors of the Guth papers appear to be members of a single committee, the SKLM commission of the German Research Academy, which is charged with reviewing food safety [301]. A senior author, Jan Hengstler, is the chair of the commission. Another author, Gerhard Eisenbrand, is the most senior member and was the decades-long chair of SKLM before Hengstler took the reins [302]. The SKLM commission appears to have been a virtual fiefdom of Eisenbrand and has had unprecedented influence in evaluating and regulating chemicals found in foods, in Germany and the European Union (EU) [303, 304].”*

*(Neurath, 2025; p. 21)*

The “Guth paper” refers to a publication in which the SKLM assessed the toxicity of fluoride (Guth et al., 2020). The fact that the authors of this publication are members of the DFG Senate Commission on Food Safety (SKLM) does not call their independence into question. The SKLM’s mandate is precisely to provide expert, evidence-based assessments on food safety issues and publications such as the cited paper are therefore a core part of its scientific function. As with all scientific committees, the SKLM has a chair to coordinate its work, which does not confer authority resembling a “fiefdom”, nor does it imply undue influence over individual members.

Furthermore, it should be noted that the SKLM does not regulate substances in food. Regulatory decisions lie with the competent national and European authorities. As part of the DFG, the SKLM’s mandate is to provide independent scientific assessments related to food safety, not to implement policy or regulatory decisions.

**Reply to personal allegations against Jan Hengstler**

1. Alleged tobacco industry funding

*“The tobacco industry seems to have financially rewarded Hengstler … with over $500,000 in research funding [343].” (Neurath, 2025; p. 24)*

This allegation is **false**. Prof. Dr. Hengstler has never received any payments or benefits from the tobacco industry. All his research funding originates from **public sources** and is fully documented on the following website: https://www.ifado.de/en/research/toxicology/funds.

2. Alleged chemical industry funding

*“By 2004, … Hengstler had moved on from tobacco industry funding to chemical industry funding…” (Neurath, 2025; p.25, 3rd paragraph)*

This statement is also **incorrect**. Prof. Dr. Hengstler has never received funding from the tobacco or chemical industries. He has also obtained no payments or funding from the “sugar industry”. All his funding sources are public and are disclosed on the website mentioned above.

3. Misrepresentation of professional correspondence

Without prior contact, Neurath accessed and published e-mails sent from Prof. Hengstler’s institutional account (Neurath, 2025; pp. 18–19). This is accompanied by the following text:

*“…the federal agency most responsible for promoting fluoridation–arranged to privately meet with the German authors in an apparent effort to get assistance counteracting the NTP systematic review (Figs. 9 and 10). The documents released by the US CDC are heavily redacted, suggesting the CDC wished to hide the details of their communications with the authors of Guth et al. [27]
Not only were the authors of Guth et al. apparently asked to secretly help the CDC counteract NTP’s systematic review of fluoride neurotoxicity, they were also chosen to peer-review an update of a New Zealand government report on fluoridation that contains numerous errors and makes conclusions almost identical to those of Guth et al. [276, 277].”*

The cited publication Guth et al. (2020) is a review of fluoride toxicity, in which Prof. Dr. Hengstler is senior author. He did not “meet” health authorities from the USA (or from New Zealand) “privately”, but rather **communicated openly via his institutional e-mail account**, as is standard practice when health authorities or other researchers contact authors regarding published work.

It is incorrect to suggest that Prof. Dr. Hengstler was asked “to get assistance counteracting the NTP systematic review” or that he intended to “hide the details of their communications”. Prof. Dr. Hengstler has no objection to disclosing the full e-mail correspondence related to the fluoride paper. Such disclosure, however, should be made in its entirety and with consent, as releasing isolated parts of the correspondence without full context could create a misleading impression. For reference, the missing text on p. 19, which was not redacted by Prof. Dr. Hengstler, but by the CDC counterparts and which was omitted in Neurath (2025) states that:

- The purpose of the meeting was “solely informational, and will not constitute any guidance, evaluation, identification, or consideration by CDC of any proposed course of action”
- “CDC’s invitation to meet should not in any way be construed to imply that CDC endorses or approves of any particular organization or its products or services, or that CDC agrees with any health claims that may be asserted”
- “any information provided during the meeting will be a matter of public record.”

In the same written communication, CDC’s Division of Oral Health offered to *“host a virtual listening session to provide an opportunity for you to discuss the findings from your analyses* [*Toxicity of fluoride: critical evaluation of evidence for human developmental neurotoxicity in epidemiological studies, animal experiments and in vitro analyses*](https://www.ncbi.nlm.nih.gov/pmc/articles/PMC7261729/) *and* [*Contribution to the ongoing discussion on fluoride toxicity*](https://www.ncbi.nlm.nih.gov/pmc/articles/PMC8241794/)*” and proposed to first present the findings from the papers listed above and “10 minutes for any clarifying questions we may have”*.

This emphasizes that the aim of this exchange was entirely scientific and solely informational. Such discussions are a common and accepted practice in the scientific community, allowing researchers and public health agencies to share data, clarify findings, and foster evidence-based dialogue.

4. Alleged “bending of science”

*“Guth et al. also use a straw-man tactic, focusing their attention on claimed weaknesses in some of the early studies but suggesting those weaknesses apply to the entire body of studies including higher-quality studies published after 2014. Guth et al. employ recognized methods of “bending science” [31]”* (Neurath, 2025; p. 20, right column)

This is **incorrect**. Each study included in Guth et al. (2021) was analyzed individually and conclusions were drawn based on the quality and strength of the evidence provided by the respective studies.

**Reply to personal allegations against Gerhard Eisenbrand**

1*.* Alleged no declaration of conflict of interest

1.1. *“Alleged numerous conflicts of interest with industry, yet he often does not declare them”* (Neurath 2025; p.21, 2^nd^ paragraph).

This statement is incorrect. The cited reference appears to refer to a declaration of interests submitted by Prof. Dr. Eisenbrand in 2012 when invited to join the EU Scientific Committee on Consumer Safety (SCCS). According to SCCS rules, a declaration of interest does not automatically constitute a conflict of interest. The declaration covered scientific collaborations dating back to 2007, including those referenced by the author. Neither the SCCS nor other authorities, such as the BfR, identified any conflicts of interest, and Prof. Dr. Eisenbrand worked with these bodies over several years in full accordance with established procedures.

1.2**.** The footnote on the same page demonstrates the negligence of facts prevailing in the Neurath statement: “*A more recent conflict of interest that was not acknowledged in Eisenbrand’s declaration for the EU was his membership in the Flavor and Extract Manufacturers Association (FEMA) committee on Food additives generally regarded as safe (GRAS)”.*

**This statement is incorrect in two respects.** First, the cited DOI refers to a 2012 publication, several years before Prof. Dr. Eisenbrand was elected to the FEMA GRAS panel. Second, in the FEMA GRAS panel he became a member of a panel to evaluates the **safety of flavoring agents**, not food additives.

1.3. Alleged “*most egregious conflict*” arising from longtime leadership of the International Life Sciences Institute (ILSI), described as an industry front group (Neurath, 2025; p. 21, last paragraph)

This claim is false. Prof. Dr. Eisenbrand’s scientific activities for ILSI Europe, including his service as President of the Board of Directors for about 5 years, not as Executive and Scientific Director, as improperly alleged, were fully transparent and publicly documented. ILSI operates under strict principles of scientific integrity and a code of ethics that explicitly prohibits lobbying. The ILSI code of ethics, publicly available from the internet^[2]^, says:

*“Principle 3. Advocacy of any kind is strictly limited to promotion of the use of evidence-based science as an aid in decision-making. ILSI does not conduct lobbying activities. “*

All scientific outputs produced with Prof. Dr. Eisenbrand’s involvement were published in peer-reviewed journals and contain only transparent, evidence-based results.

2. Alleged “bending of science”

This allegation has already been addressed and refuted in Prof. Dr. Hengstler’s response.

**Invitation to an Open Scientific Discussion**

Rather than continuing a personal exchange, we invite *Environmental Health* and all interested parties to return the discussion to the **scientific questions at its core**. We welcome an **open, evidence-based scientific dialogue.**

1. <https://www.dfg.de/resource/blob/168560/72-01-en.pdf>
2. https://ilsi.org/wp-content/uploads/2023/01/ILSI-Code-of-Ethics-1022.pdf
